# Enhanced endoscopic transillumination enables precise tumor delineation in laparoscopic-endoscopic cooperative surgery for duodenal neuroendocrine tumor

**DOI:** 10.1055/a-2792-9422

**Published:** 2026-02-24

**Authors:** Huan He, Yuting Tian, Mingxin Ye, Xiaowei Tang, Wenguang Fu, Muhan Lü, Lei Shi

**Affiliations:** 1 556508 Department of Gastroenterology, Biliary-Pancreatic Center, The Affiliated Hospital, Southwest Medical University, Luzhou, China; 2 556508 Department of Gastroenterology, The Affiliated Hospital, Southwest Medical University, Luzhou, China; 3 556508 Department of General Surgery (Hepatobiliary Surgery), Biliary-Pancreatic Center, The Affiliated Hospital, Southwest Medical University, Luzhou, China; 4 556508 Metabolic Hepatobiliary and Pancreatic Diseases Key Laboratory of Luzhou City, Academician Workstation of Sichuan Province, The Affiliated Hospital, Southwest Medical University, Luzhou, China; 5 556508 Department of Endoscopic Medicine, The Affiliated Hospital, Southwest Medical University, Luzhou, China

**Fig. 1:**
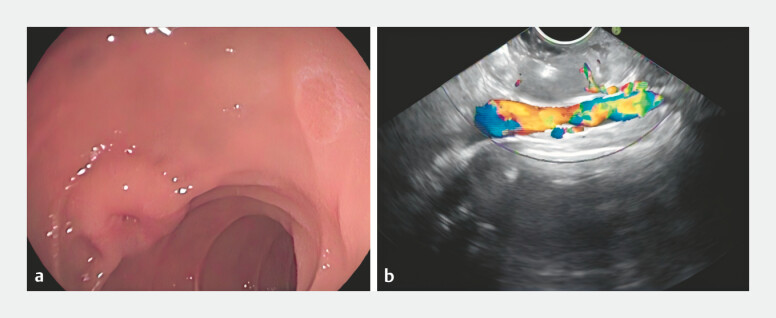
**a** Gastroscopy identified a protruding lesion in the descending duodenum. **b** Endoscopic ultrasound revealed a hypoechoic mass, measuring approximately 2.0 × 1.9 cm, within the muscularis propria.

A 21-year-old woman presented with melena. Gastroscopy revealed a protruding lesion in the descending duodenum. Endoscopic ultrasonography showed a ~2.0 × 1.9 cm hypoechoic mass originating from the muscularis propria ( Fig. 1 ). Given the deep location, laparoscopic-endoscopic cooperative surgery (LECS) was selected for full-thickness resection.

**Fig. 2:**
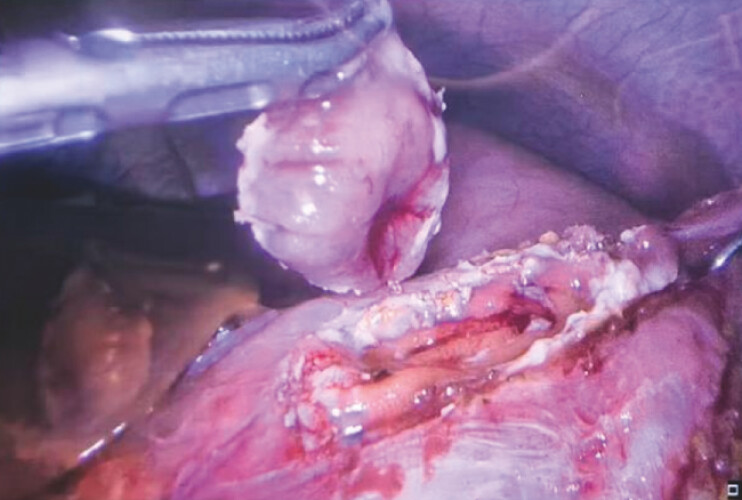
The tumor was completely resected laparoscopically.

**Fig. 3:**
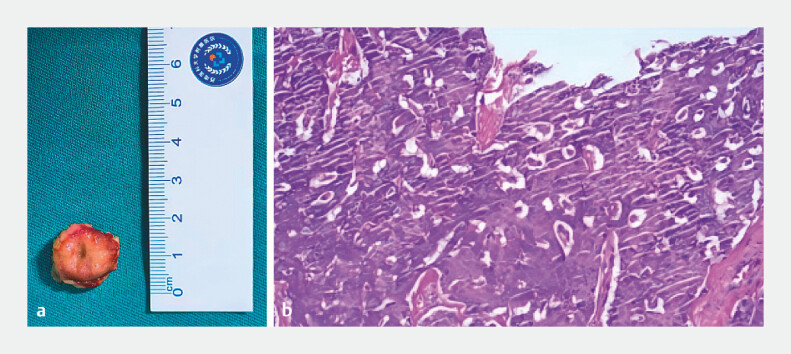
**a** A macroscopic view of the 19 × 16 × 6 mm specimen. **b** H&E staining reveals nests of epithelioid cells in the lamina propria, consistent with a neuroendocrine tumor, G1.

After Kocher maneuver, the gastroscope was positioned at the tumor. Endoscopic enhanced transillumination (Olympus, TRANSILLUM Mode) clearly illuminated the intestinal segment, outlining the entire tumor contour and margins ( Video 1 ), enabling precise co-localization. The resection area was marked 3 mm outside the border. Following laparoscopic resection ( Fig. 2 ), the duodenum was sutured in layers. Final gastroscopy confirmed no bleeding, normal papilla, and no stenosis. The operative time was 40 minutes with minimal blood loss. Pathology confirmed an R0 resection of a 19 × 16 × 6 mm NET G1 ( Fig. 3 ). The patient recovered uneventfully.

Enhanced endoscopic transillumination provides clear delineation of tumor margins in a duodenal NET, facilitating a conservative and precise resection via the LECS approach. LECS, laparoscopic-endoscopic cooperative surgery; NET, neuroendocrine tumor.Video 1

LECS is a feasible, safe approach for duodenal tumors 1 . However, determining serosal-side resection margins is challenging, and excessive resection risks stenosis. Although existing transillumination techniques have been documented 2 , they often provide suboptimal illumination for defining the complete tumor margins. This case demonstrates that the novel application of enhanced endoscopic transillumination enabled precise tumor delineation without compromising laparoscopic lighting, thereby facilitating a conservative yet precise resection and establishing a safe and effective visualization strategy for duodenal neuroendocrine tumors.

Endoscopy_UCTN_Code_TTT_1AT_2AD
